# Integration of Metabolomic and Other Omics Data in Population-Based Study Designs: An Epidemiological Perspective

**DOI:** 10.3390/metabo9060117

**Published:** 2019-06-18

**Authors:** Su H. Chu, Mengna Huang, Rachel S. Kelly, Elisa Benedetti, Jalal K. Siddiqui, Oana A. Zeleznik, Alexandre Pereira, David Herrington, Craig E. Wheelock, Jan Krumsiek, Michael McGeachie, Steven C. Moore, Peter Kraft, Ewy Mathé, Jessica Lasky-Su, on behalf of the Consortium of Metabolomics Studies Statistics Working Group

**Affiliations:** 1 Channing Division of Network Medicine, Brigham and Women’s Hospital and Harvard Medical School, Boston, MA 02115, USA; mengna.huang@channing.harvard.edu (M.H.); hprke@channing.harvard.edu (R.S.K.); ozeleznik@bwh.harvard.edu (O.A.Z.); remmg@channing.harvard.edu (M.M.); jessica.su@channing.harvard.edu (J.L.-S.); 2 Institute for Computational Biomedicine, Englander Institute for Precision Medicine, Department of Physiology and Biophysics, Weill Cornell Medicine, New York, NY 10021, USA; elb4003@med.cornell.edu (E.B.); jak2043@med.cornell.edu (J.K.); 3 Department of Biomedical Informatics, College of Medicine, The Ohio State University, Columbus, OH 43210, USA; jalal.siddiqui@osumc.edu (J.K.S.); ewy.mathe@osumc.edu (E.M.); 4 Department of Genetics and Molecular Medicine, University of Sao Paulo Medical School, Sao Paulo 01246-903, Brazil; alexandre.pereira@incor.usp.br; 5 Department of Internal Medicine, Wake Forest School of Medicine, Winston-Salem, NC 27101, USA; dherring@wakehealth.edu; 6 Division of Physiological Chemistry 2, Department of Medical Biochemistry and Biophysics, Karolinska Institute, 171 77 Stockholm, Sweden; craig.wheelock@ki.se; 7 Division of Cancer Epidemiology and Genetics, National Cancer Institute, Rockville, MD 20850, USA; Steve.Moore@nih.gov; 8 Department of Epidemiology, Harvard School of Public Health, Boston, MA 02115, USA; pkraft@hsph.harvard.edu

**Keywords:** multi-omic integration, systems biology, epidemiology, study design, integrative analysis

## Abstract

It is not controversial that study design considerations and challenges must be addressed when investigating the linkage between single omic measurements and human phenotypes. It follows that such considerations are just as critical, if not more so, in the context of multi-omic studies. In this review, we discuss (1) epidemiologic principles of study design, including selection of biospecimen source(s) and the implications of the timing of sample collection, in the context of a multi-omic investigation, and (2) the strengths and limitations of various techniques of data integration across multi-omic data types that may arise in population-based studies utilizing metabolomic data.

## 1. Introduction

Long considered a ‘link between genotype and phenotype’ [1], the metabolome can offer a unique view not only into the catalogue of end products of biochemical reactions underlying complex traits, but also into the potential environmental contributors which may interact, directly or indirectly, with the small molecules comprising the metabolome [2,3]. In this sense, as both endogenous and exogenous perturbations can be captured by metabolomic snapshots, the metabolome can serve as a rich resource for identifying biomarkers for the prediction, prognosis, diagnosis or subtyping of human disease. If, however, the goal of a study is to identify or estimate biological mechanisms and relationships, the study of metabolomics alone may produce limited insights.

**Figure 1:**
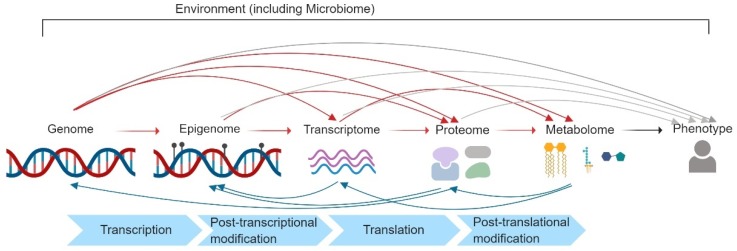
A systems biology view of complex trait etiology. Red arrows reflect all potential causal mechanistic pathways that may be captured by the metabolome assuming a central dogma framework. Gray arrows depict biological pathways that do not act through changes to the metabolome. Blue arrows depict potential sources of reverse causation, or mechanisms that involve time-dependent feedback between omics and do not strictly adhere to the central dogma; the arrows depicted here are non-exhaustive of all potential reverse causation/time-dependent paths. The environment is depicted as a potential force across all of the omic stages; the microbiome is included as a component of the environment, but does not necessarily exert its effects across all omic stages. Image made with Biorender.

**Figure 2:**
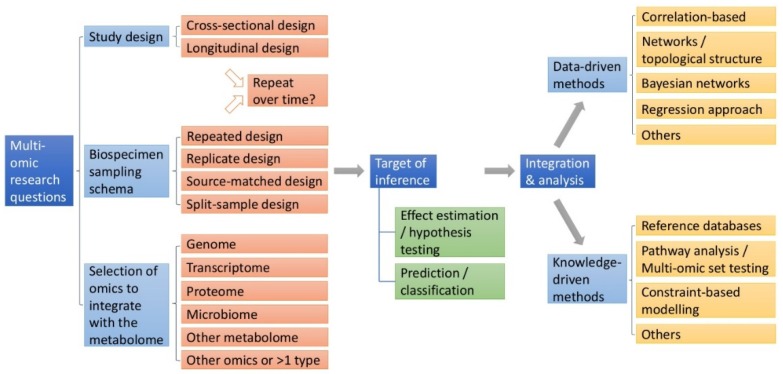
Framework for multi-omic study design and analysis. Explicitly defined research questions in a multi-omic investigation should inform three aspects of the study: study design, biospecimen sampling schema, and selection of additional omic type(s) to be integrated with the metabolome. Given the target of inference, be it effect estimation / hypothesis testing, prediction / classification, or both, we may choose appropriate methods for integration and analysis.

The number of studies integrating multiple omic data types continues to grow, increasingly demonstrating that including metabolomic information can contribute important functional insight into the interactions between the preceding omic levels (Figure 1), in addition to revealing influences exerted by the environment [2]. How these inter-omic relationships are revealed, however, necessarily depends on the study design used to recruit subjects, source and collect biosamples, and which omics are measured [4]. While current integrative studies are frequently limited by convenience with respect to the availability of multi-omic measurements, the increasing turn towards systems biology approaches warrants both careful consideration of how complex layers of omics should be integrated and thoughtful discussion regarding the limits of inference exerted by different study designs. Previous review articles on multi-omics integration have focused on its advantages over single-omic investigation in elucidating disease mechanism [5], potential impact on medical actionability [6], and experimental design, analytical methods, tools, and challenges [7], while discussion of the importance of epidemiologic study design and its implications in the context of integrative omics has been inadequate. Therefore, in this review we discuss (1) epidemiologic principles of study design, including selection of the biospecimen source(s) and the implications of the timing of sample collection, in the context of a multi-omic investigation, and (2) the strengths and limitations of various multi-omic data integration techniques that have been used in population-based studies using metabolomics data. The general outline of the multi-omic investigative framework we propose is illustrated in Figure 2. 

## 2. Study Design and Biosampling Design

### 2.1. Study Design

The kinds of inferences that can be drawn when integrating multiple omics are critically informed by study design. In addition to the selection of study participants, study design formally structures precisely when information about risk factors, intermediate processes, and outcomes is to be collected throughout the course of a study, thus naturally defining the kinds of inference that are possible. The target of inference, or the scientific question that is asked, is clearly established in the study design—for example, a study in which vitamin D levels and plasma metabolomics are collected at one time point in childhood can only point to statistical associations between the two as vitamin D levels and plasma metabolomics were measured simultaneously. On the other hand, if the vitamin D levels are measured prior to collection of the plasma for metabolomic analysis, investigators may aim to estimate the average causal effect, or lack thereof, of vitamin D on metabolite levels within the study sample over the specific period of time between the two measurements. The addition of more omic levels will make the study design and inference more complicated. For the remainder of this section, we will proceed with integrative examples using two omic data types, one of which is metabolomics. In the following examples, we will also refer to the omic type analyzed in addition to metabolomics as “Omic 1”. Although we use omic examples combining only two data types, these ideas are generalizable to more complex settings such as in studies incorporating three or more omic types. 

#### 2.1.1. Cross-Sectional Study Design

When information about a risk factor or exposure of interest is collected at the same time as an outcome or phenotype of interest, the study design is a cross-sectional design. Many multi-omic investigations are cross-sectional in nature with samples collected at a single time point, often largely due to limited availability of data or post-hoc additions to existing studies. The multi-omic cross-sectional study will collect clinical data, and biosamples to assess metabolomics and Omic 1 at the same time. The target of inference may be with respect to the joint associations between (1) the phenotype of interest and the two omics or (2) the specific associations between the two omics directly. The major limitation of this study design is that true causal effects between the exposure of interest, Omic 1, and the metabolome cannot be estimated, but rather associations between the omics and phenotype are revealed. One important exception to the above is in the context of genetics; in general, genetic variation can reasonably be expected to temporally precede a given measurement of the metabolome. 

#### 2.1.2. Longitudinal Study Designs

In longitudinal study designs, exposures are collected prior to an outcome or phenotype of interest. In these designs, the exposure and/or the outcome may be one of the levels of the ‘omics’ themselves. In general, there are three configurations of longitudinal multi-omic metabolomic analyses: (1) metabolome as the outcome, (2) metabolome as the exposure, and (3) metabolome as the mediator between a risk factor of interest and an outcome of interest. In the following, we discuss the importance of the temporal ordering of data collection as an initial framework for answering research questions, especially those that are causal in nature. However, we emphasize that such temporal ordering provided in formal study design planning is only the first requirement in the process of inferring causality. Many more assumptions must hold in formal causal inference analyses, such as no residual confounding assumptions, which are beyond the scope of this review, but which have been discussed at great length elsewhere [8,9].

**Figure 3:**
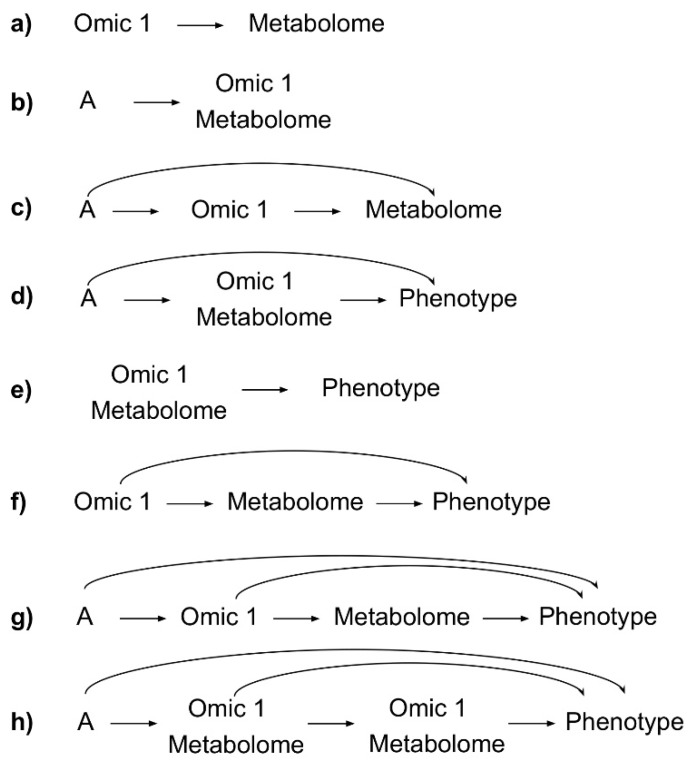
(**a**–**h**) Examples of multi-omic research questions that can be addressed in a multi-omic, longitudinal study design, represented as directed acyclic graphs. Time flows from left to right, to indicate distinct points of data collection. A = Exposure.

*Metabolome as the Outcome.* In a multi-omic longitudinal study with the metabolome as the outcome, several hypotheses may be addressed. If Omic 1 was collected and measured prior to the metabolome, and the interest is in establishing the relational context between them, the effect of variation in Omic 1, as the exposure, on variation in the metabolome, as the outcome, would be the target of inference (Figure 3a). Alternatively, a clinical risk factor might be the exposure of interest (Figure 3b, exposure denoted A). In this case, the study of Omic 1 and the metabolome would necessarily depend on when the collection of the samples that generated each omic data type occurred. If the samples for Omic 1 and metabolomics were collected simultaneously, then the valid research question would be framed around the effect of exposure A on Omic 1 and the metabolome jointly. When Omic 1 can be reasonably assumed to be stable over time in this situation, Omic 1 may also be treated as the mediator between A and metabolome in the analysis stage. More explicitly, if the sample collection for Omic 1 precedes the sample collection for metabolomics, the study of a potential causal path from the exposure A, to Omic 1 (the mediator), to the outcome, metabolome, would be appropriate (Figure 3c). However, if Omic 1 is highly variable in nature, its measurement at one single time point between A and the metabolome may not be adequate to capture the causal effect, or it may lead to substantial uncertainty around the estimates.

*Metabolome as the Exposure.* The most abundantly observed use-case of metabolomics is for the prediction of disease risk. If the assumption that the metabolome is the most proximal omic to phenotype holds, then the use of the metabolomics to identify biomarkers of disease is a logical approach. However, if the goal is to identify biological mechanisms or estimate the effect of metabolites on disease, rather than to classify or predict, an integrative approach that jointly assesses the effect of Omic 1 and metabolomics may be sensible (Figure 3e). In this design, the joint relationship between an exposure A and the metabolome on a disease outcome may also be assessed. Of note, case-control studies with omic data and disease status collected concurrently often assume that the direction of association/causality is from omics to disease in hypothesis testing, though the presence of reverse causation may invalidate this assumption. The goal of this type of design is better described as an assessment of the variation in omic measurements comparing diseased vs non-diseased states. Subclinical manifestations of certain diseases may also affect both Omic 1 and metabolome even when they are measured from samples collected prior to disease diagnosis. For studies where subclinical phenotypes are plausible or likely, the discussion of results should acknowledge that observed variation in omic measures may be a manifestation of the disease itself. 

*Metabolome as a Mediator.* The metabolome is perhaps best biologically characterized as a mediator, or a non-mediating marker, of effects from an exposure to a phenotypic outcome of interest (Figure 1). In the case where the study question hypothesizes a mediating role for the metabolome, the exposure may be defined as an environmental factor or even an earlier omic state. When it can be clearly established that Omic 1 temporally and/or causally precedes the metabolome, and both Omic 1 and the metabolome precede the outcome, we may attempt to establish flow of causality/associations from Omic 1 (e.g., genetic variation, or gene expression) to the metabolome, and to a phenotype (Figure 3f). If Omic 1 and metabolomics are measured from samples collected at the same time point, they could also jointly mediate the effect of a preceding exposure (A) on the phenotype of interest (Figure 3d). Furthermore, in the example of Figure 3g, a decomposition of effects is obtainable if A is collected prior to Omic 1, Omic 1 is collected prior to metabolomics, and metabolomics is collected prior to disease manifestation. With longitudinal designs collecting Omic 1 and metabolomics at multiple time points between an exposure of interest (A) and development of disease (Figure 3h), the degree to which Omic 1 and metabolomics jointly mediate the effect of A on the phenotype over time may also be evaluated.

### 2.2. Biospecimen Sampling Schema

**Figure 4:**
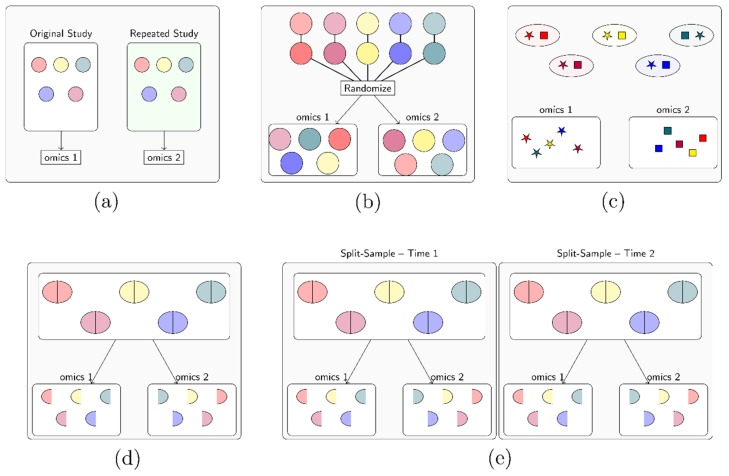
Common study designs in multi-omics studies. (**a**) Repeated design; (**b**) Replicate design; (**c**) Source-matched design; (**d**) Split-sample design; (**e**) Longitudinal split-sample design.

In addition, to the epidemiologic study designs discussed above, integrating data from multiple omics also involves different experimental designs pertaining to sampling, potentially at different time points and/or from different biological sources. In the designing stage of a multi-omic study, investigators should plan for adequate laboratory expertise in sample collection, handling, and processing for different omic types in potentially different biospecimens. Here we focus on considerations in the epidemiological design of sampling. Four common types of sampling schemas, in multi-omics study were discussed by Cavill et al. (Figure 4) [4]. 

In the repeated study design (Figure 4a), the original study generates data for one omic type, and sample collection is repeated on the same subjects at one or more time points distinct from the first. When integrating other omic data types with metabolomics, in addition to analytic considerations for within-person correlation and batch effects (i.e., technical variation attributable to non-biological, sources such as those arising due to changing laboratory conditions or processing samples at different times [10]), the extent to which the omic data varies over time and cell/tissue types, and how this may impact data integration should be considered: for example, genetic variation is stable, but many other omics, such as gene expression and DNA methylation, are not. It follows that whether it is sensible to integrate these data with metabolomics at selected study time points is thus context dependent. For example, metabolomics from two or more different time points can be integrated to examine how profiles change over the course of a given period (e.g., early life/developmental periods), while integrating transcriptomic data with metabolomic data collected five years later would make more sense if there was a reasonable belief that the transcripts or transcriptomic profiles of interest could be expected to persist, or have effects on metabolites which persist, years after the initial measurement. 

The other three study designs all involve sampling at the same time point on a defined set of study subjects. The replicate-matched study design (Figure 4b) is frequently used in in vitro studies when the protocols of obtaining two or more types of omic data require different processing techniques. Two or more replicates of the same samples are collected and prepared at the same time for data generation. The replicates should be randomized for measurement by different omic technologies to minimize potential batch effects in sample collection and preparation. In other situations, investigators may wish to integrate omics data from different parts of the same biological system, for instance plasma and stool metabolomics, or blood transcriptomics and urinary metabolomics. This approach can be defined as a source-matched study design (Figure 4c) when the samples are collected at the same time on the same set of study subjects. In the split-sample study design (Figure 4d), samples obtained during one single collection (sometimes also mixed, e.g., to achieve homogeneity in urine samples) are split into two or more sections for different assays to obtain a multitude of omics data, which is more commonly seen in vivo studies. Aliquots could also be made and stored for validation or for future enhanced (e.g., single cell) measurements. 

The replicate-matched, split sample, and source-matched designs may also be repeated longitudinally (for an example see Figure 4e), although careful considerations are required in the statistical analyses of such complex data. A longitudinal design is necessary to capture trends over time for most types of omic data. For transparency, the temporality among the multi-omic data as well as other dependent or independent variables involved in the analyses should be clearly stated when reporting results. 

When integrating data from multiple omics, whether they come from the same sample source influences the inference we will be able to make. In a split-sample design, the data come from the same tissue and are concurrent, which can enable the exploration of disease mechanisms within that particular tissue at the specific time point of sample collection. For example, we may seek to characterize molecular networks in a disease state using omic data obtained from the same tissue, as in a study of lung function in childhood asthma integrating transcriptomic and metabolomic data from the same blood samples [11]. The source-matched design illustrates a situation where different omics data come from different sample sources. We may establish systematic networks across tissue types with such multi-omic data, which could be advantageous in the study of human health conditions impacting more than one type of tissue or organs. Furthermore, evaluating different sample sources may also corroborate and solidify findings. Specifically, if constituents of Omic 1 and metabolites of interest are measured in different sample sources yet reflect similar biological pathways and mechanisms, then similarities between individual omics can provide an alternative form of validation, or lend greater credibility to the findings in a study. One example of this approach is the evaluation of genes in kidney tissue and metabolites in plasma and urine, uncovering the role of the citric acid cycle in chronic kidney disease [12].

In review, study designs involving different time points and/or different sample sources can be categorized into three categories: (1) same sample sources collected at multiple time points, (2) different sample sources collected at the same time point, (3) different sample sources collected at different times. Of course, while the concepts above may generally hold, exceptions to these frameworks may still arise: for example, in a birth cohort study where metabolomics data are obtained from pairs of mothers and offspring, possibly longitudinally at multiple time points, and the associations between maternal metabolome and the children’s metabolome are of interest.

### 2.3. Selection of Additional Omic Type to Integrate with Metabolomics

#### 2.3.1. Genome and Metabolome

Studies integrating metabolomic data with germline genomic information are some of the most commonly conducted integrative metabolomics studies to date. In these studies, concerns with respect to temporality are ameliorated—by design the genome must precede the metabolome. Additionally, although metabolite levels may vary by tissue type; the genome does not, making sample selection more straightforward. Researchers are increasingly using the metabolome to explore the downstream functional implications of single nucleotide polymorphisms (SNPs), including in relation to disease-associated SNPs. However, the biospecimen source in which the metabolomic data are measured is of crucial importance for biological interpretation. While blood/plasma samples reflect systemic metabolite levels, integrating metabolomics from all tissue types, e.g., results from studies measuring metabolites in hair, nails or other tissues, will require interpretations that account for the specific exposure(s) captured by each sample source. Organ-specific tissue/biofluid samples measure local levels of metabolites and thus may reveal tissue-specific SNP-metabolite associations, but are more difficult and sometimes unethical to collect. Finally, although we have already established that the genome precedes the metabolome, it remains critically important to determine if there is a particular window of time (i.e., sensitive periods) during which genetic risk factors of interest might exert their effects on metabolites downstream (for example in the case of developmental diseases of genetic origin such as phenylketonuria).

Metabolite quantitative trait loci (metaboQTL) can be considered analogous to expression QTL (eQTL) [13,14]; they are SNPs that are associated with levels of one or more metabolites. As such, metaboQTLs can help to better understand the biological processes governing biological systems by identifying the downstream effects of SNPs on the metabolome. A number of metabolomic genome-wide association studies (mGWAS) have been conducted to date, revealing the complex balance between genetic and environmental influences that determines metabolite levels. Overall, the variation in blood metabolite levels explained by genetic variants has been estimated to range from 2% to 63% [15]. Given the role of the metabolome as an intermediate between genotype and phenotype, metaboQTLs can provide insights into the biochemical mechanisms driving gene-disease associations by identifying the metabolites and metabolic pathways affected by the genetic variants. The number of known metaboQTLs continues to increase, with efforts to catalogue findings available through resources such as the ‘Metabolomics GWAS server’ (http://mips.helmholtz-muenchen.de/proj/GWAS/gwas/). A natural extension of single metaboQTLs is represented by efforts to associate metabolite groups/pathways with SNPs [16,17] which may increase the statistical power of the analysis by reducing the number of tested hypotheses.

The concept of metaboQTLs can be extended to the idea of genetically informed metabotypes (GIMs) which encompass an ensemble of metaboQTLs, that often cluster in high linkage disequilibrium and associate with the same metabolites or metabolites in the same class, pathway or process, thereby having a coordinated effect on phenotype [18]. These GIMs tend to have large effect sizes and to manifest as complex traits [18]. It should be recognized that these findings are-based largely on data-driven associations, rather than on biological knowledge. Integrative methods incorporating what is known about the genome and metabolic reactions may lead to greater insight into specific mechanisms of disease by focusing on particular putative disease pathways, although our knowledge of such pathways is admittedly far from complete. 

There are several other limitations inherent to studies integrating genetic and metabolomics data. First, biological interpretation of metaboQTLs and GIMs can be challenging. They are most interpretable when they involve SNPs that influence gene expression in genes encoding enzymes that catalyze biochemical reactions [18]. Less emphasis has been placed on intronic SNPs, and the concept of cis- versus trans- metaboQTLs remains to be explored. Investigations in tissues closest to the site of pathophysiology are often not available. To date, the majority of studies have focused on the blood or urine metabolomes. MetaboQTLs in other tissues or biofluids are vital to fully comprehend the genotype-metabolome relationship on a whole system level. Similarly, gender-specific integration may be necessary, as evidenced by the work in gender-specific whole-body metabolism reconstructions. Finally, genetic variation explains only some of the variance in metabolite levels and the importance of the environment cannot be overlooked. 

#### 2.3.2. Transcriptome and Metabolome

Integrating gene expression with metabolomics can generate hypotheses on how these metabolic phenotypes are regulated, which could in turn elucidate targetable functional mechanisms to generate a desired phenotype. Integration of metabolomic and transcriptomic data can thus increase our understanding of the factors affecting metabolite levels, regardless of whether or not the metabolite identity is known. Application of this integrative approach in diseases, including cancer, has highlighted key disease-related metabolic functions and pathways [11,19,20,21,22,23]. 

When transcriptomic and metabolomic data are acquired from the same individuals and sample sources, the interplay between metabolite and gene level can be directly evaluated, yielding molecular networks that reflect molecular mechanisms. One note of caution is that gene-metabolite relationships found may not imply causation. The relationship between genes and metabolites is very complex, involving non-linear reaction kinetics mechanisms, enzyme activity and substrate affinity, metabolite-metabolite negative or positive feedback loops that regulate metabolite levels, and post-translational modifications [24,25]. These complex relationships cannot be directly evaluated with the resolution of the data (e.g., relative measurements of metabolite and gene-expression levels as opposed to flux analyses) that is typically acquired in larger-scale epidemiologic studies. Nonetheless, this approach has been successfully applied to asthma [11], cancer [22,26], and preeclampsia [27], and has yielded important knowledge of molecular mechanisms that drive a given phenotype at a population level. 

When data are collected in the same sample source (e.g., blood) at multiple time points (repeated design in same sample source), it may be possible to evaluate how Omic 1-metabolome relationships and associated pathways change over time, and whether these changes relate to a phenotype (e.g., disease progression, onset of infection, etc.). Examples of this design in epidemiological studies are sparse as it requires both deep (large-scale metabolomic and transcriptomic profiling) and broad (in many samples at different time points) coverage. Nonetheless, this design has been successfully applied to uncover metabolic pathways associated with body weight change [28], including lipid and amino acid metabolism, insulin sensitivity and blood cell development and function. Other applications include the analysis of temporal patterns of gene-metabolite networks in one individual, the integrative personal omics profile [29], and in model systems [30]. Notably, this design has great potential for evaluating human health metrics over time using wearable biosensors [31], and for identifying putative metabolite biomarkers that reflect disease-specific alterations in other omics.

Alternatively, data can be collected in different sample sources from the same individuals at the same time point. Rather than evaluating possible direct relationships between genes and metabolites, investigators can assess whether one measurement can act as a putative biomarker for another. For example, strong correlations between gene-expression levels in diseased tissue and urinary metabolites provides preliminary evidence that these urinary metabolites could act as putative biomarkers of the underlying disease, and can thus be assessed in a much less invasive manner. It is, however, necessary to establish the specificity of the urinary markers for the disease in question. 

#### 2.3.3. Proteome and Metabolome 

The proteome refers to all of the proteins expressed in a given cell, tissue, or organ, but its complexity increases dramatically due to the formation of protein complexes and interactions as well as subcellular localization. The proteome is perhaps the omic type most closely linked to the metabolome, given that many metabolite levels are directly regulated by proteins/enzymes involved in their metabolic pathways. However, studies integrating metabolomics and MS-based proteomics are not as common as those integrating metabolomics with transcriptomics or with genetic variation. The paucity in integration studies is mostly due to technical challenges and cost in conducting large-scale proteomics and metabolomics relative to the high-throughput technologies available for genetic data. Nonetheless, new technological developments are rapidly changing this scenario and projects such as the Human Protein Atlas are dramatically increasing our knowledge by mapping all human proteins in cells, tissues and organs [32,33,34]. Advances in top-down proteomics are providing increased detail in the myriad of different human proteoforms [35,36] (a proteoform includes all possible protein products from a single gene including genetic variation, alternate splicing, and post-translational modifications [37] and presents an interesting target for investigating protein-metabolite interactions). 

Similar to metabolomics, several different platforms and sample preparation methods can be employed to derive a broad overall representation of proteins in complex biological fluids and tissues. Targeted proteomic assays have the needed sensitivity to quantitatively detect up to hundreds of different proteins. Untargeted shotgun approaches are able to detect thousands of different proteins/peptides. However, depending on the technique used, they do not present a robust dynamic range (generally orders of magnitude less than that seen in complex biological fluids), and are sub-optimal for absolute or relative quantification. Recently, however, aptamer-based targeted proteomic panels have become available and have allowed for an unprecedented combination of sensitivity, scalability and wide protein molecules representation [38]. This new technology has allowed the derivation of large-scale proteomic data from thousands of samples from the same study, without the need for laborious sample preparation and prolonged processing time. A major driver of these efforts will be the ability to perform multi-omic analyses from the same sample, which has been successfully done for metabolomics and proteomics [39,40].

An interesting advance in proteomics has been the advent of the adductomics approach. This subtype of proteomics is focused on the small molecule chemical adducts of proteins, in particular cysteine residues [41]. This method has been proposed for applications in exposomics to measure the Environment portion shown in Figure 1 [42]. The combination of adductomics with metabolomics can provide a measure of the interaction between both the external exposome and the systemic alterations in oxidative stress. Previous work has provided insights into cancer induction following benzene exposure [43]. It will therefore be of significant interest to integrate adduct-based proteomics data with metabolomics to achieve greater understanding of the exposome. 

Traditional efforts to integrate proteomic and metabolomic data have often focused on combined pathway mapping. While informative and providing increased molecular resolution, this approach does not exploit the increased statistical power that can be achieved through more integrative methods [44]. The utility of combing the molecular information to increase our understanding of biochemical and disease processes is clear. For example, integrated proteomics and metabolomics showed the importance of circulating lipids and the coagulation cascade in the progression of septic shock [45]. Although combining these data structures is biologically sensible, the multitude of different approaches so far proposed highlight the complexity of the endeavor. A typical example of the metabolite-proteomic integration is the use of genome-scale metabolic reconstruction models as the theoretical framework, and both metabolomics and proteomics data as inputs to flux balance analysis [46]. While the increased molecular insight due to the acquisition of metabolomics and proteomics data is evident, there is still a concomitant need in integrative statistical methods that move beyond simple pathway mapping (e.g., KEGG).

The logical connection between proteins and their metabolic products makes integrated proteomics and metabolomics an important component of a multi-omic strategy. Given the current limitations in both proteomics and metabolomics, the development of integration strategies will rely on both technological developments that will increase sample representation and information content. In addition, it is important to highlight that not all proteomics or metabolomics datasets are equally informative for understanding disease processes. For example, in the case of obstructive lung disease, it is naturally more informative to perform the analyses in biosamples taken from the lung vs circulatory profiles. This becomes important for the development of integrative models and the resulting molecular resolution and statistical power. It was previously shown that proteomics and metabolomics from bronchoalveolar lavage fluid were more informative for discriminating healthy smokers vs smokers with chronic obstructive pulmonary disease than the corresponding blood profiles [44]. This is of particular relevance in the multi-omic framework in Figure 2, because this type of sampling may not be suitable for some study designs (e.g., it is not ethical to perform longitudinal bronchoalveolar lavages). Accordingly, study design will be an eventual compromise between breadth of molecular information, temporal interactions between omic profiles and experimental feasibility. The advent of novel modeling approaches in combination with new analytical developments will enable the field to leverage these informative large-scale data resources.

#### 2.3.4. Microbiome and Metabolome 

Many biological systems, including humans, co-exist with their microbiomes, and the two interact cooperatively in sustaining functions such as defense, metabolism, and homeostasis [47]. Here we use the term “microbiome” to refer to both the microbial taxonomic profiles and the collective multi-omics (e.g., metagenome, metatranscriptome, and metaproteome) of the microorganisms that reside in an anatomical site. It is increasingly recognized that the human microbiome plays a significant role in human health conditions and disease progression [48]. In addition to producing metabolites of its own, the microbiome may also influence gene expression, immune response, and metabolism in the host [47,48]. Comparing metabolites in the plasma of germ-free and conventional mice, around 10% of all features had significant differences, and hundreds were present only in conventional animals, hinting at the potential effects of the microbiome have on host metabolism [49]. In humans, specific microbial species have been identified to drive the association between the biosynthesis of essential branched-chain amino acids and insulin resistance [50]. Therefore, a natural research question is to explore the relationship between the microbiome and metabolome, interactions between the microbiome and the host, as well as their integrative effects on health outcomes.

Taxonomic variation and functional variation of the microbiome across different anatomical sites of a biological system have profound implications in the interpretation of microbiome and metabolome relationships, which also depends on the matrix of metabolomic data. Samples may be collected from a common biological source at the same time (for example within the same study visit), such as the case of fecal microbiome and metabolome [51,52], or collected from different biological sources, for example gut microbiome and serum/plasma metabolome [53,54]. Integrating omic data from different levels of biological regulation may provide important insights into the overall picture and hierarchical architecture of multi-organ disease pathophysiology [55].

Both the metabolome and microbiome vary in response to factors such as diet, environmental exposures, developmental stages, aging, and changes in health status [47], which adds to the complexity of data integration and interpretation of analysis results. A short interval between microbiome and metabolome measurements can help establish temporality between the two omics, but their associations may be influenced by time-varying factors that change during the interval. Longitudinal designs with repeated sample collections where multiple omics data are generated at each time point may be the most desirable study design to capture long-term relationships and changes. Nevertheless, confounding (possibly time-varying) should be properly addressed for valid estimation of effects/associations. Another potential advantage of integrating longitudinally assessed multi-omics lies in the prediction and classifications of clinical features, including disease subtyping given that appropriate analytical approaches are applied. In a recent publication using multi-omic data to characterize biological changes during pregnancy and predict gestational age, blood, vaginal swabs, stool, saliva, and tooth/gum samples were collected at multiple time points during pregnancy, which were measured for cytokines, proteome, metabolome, microbiome, and single-cell characterization of the immune system in predicting gestational age [56]. 

#### 2.3.5. Metabolome and Metabolome

In addition to integrating metabolomic data with other omic data types, multiple metabolomes can also be integrated together. While still uncommon, large cohorts are beginning to generate metabolomic data at multiple time points and/or from multiple sample sources that can be integrated together [57]. Metabolome-metabolome integration using multiple sample sources (e.g., plasma, urine, stool, and exhaled breath) is easiest when the data are generated at a single time point in which case, it is possible to directly assess the relationship between individual metabolites from multiple sample sources. The optimal study design for multi-source metabolome integration would therefore collect the different sample types at the same time or as temporally close as possible to accurately evaluate the interrelationships between these metabolomes. These interrelationships between metabolites from different sample sources is complex, because physiological regulation may modify a metabolite, such as by transforming it into a waste product en route to excretion, and also because some types of metabolites are missing from certain matrices, e.g., many lipid subclasses are not present in urine, even though they are prevalent in stool and plasma and vice versa. However, in some cases the level of a metabolite from one biological matrix will directly impact the level of that metabolites in another biological matrix. For example, high glucose levels in the blood will often result in the presence of glucose in the urine. Since cohorts often have only one type of biospecimen, it is useful when metabolites correlate strongly across multiple sample sources, as it allows more flexibility in the sample types that can be used for epidemiologic investigations.

Collecting at multiple time points from the same sample source can generate longitudinal metabolomic profiles that enable the identification of time-dependent disease processes. With this study design, the metabolomic data would ideally be generated within the same laboratory, using the same protocols, so that inter-laboratory variation does not interfere with the ability to track changes over time. When metabolomic data are generated at multiple time points, it is imperative to plan accordingly and include pooled quality control (QC) samples from all of the included times points as well as reference standards (e.g., National Institute of Standards and Technology Standard Reference Materials). Ideally, a subset of samples from the initial time point may also be rerun at the additional time points. 

Combining metabolomic data from multiple cohorts represents another form of integration. Due to the relative nature of untargeted metabolomics, direct data integration is most often not possible as the data are often generated using different laboratories, instruments, platforms, libraries, etc. Therefore, integration across cohorts most often occurs in the form of replication, validation, and/or meta-analysis of association findings.

Apart from the above-mentioned omic types, other omic data, for example the epigenome, the miRNAome, or the exposome, may also be integrated with metabolomic data. While the epigenome and miRNAome generally exert their effects on the genome or the transcriptome, the exposome may have direct impact on all other omics discussed above. These principles and considerations are generalizable to any studies integrating additional omic types with the metabolome.

## 3. Integration Paradigms and Analytic Approaches

Several useful integration paradigms in the analysis of multi-omic data include considerations of subject level data, analytic procedure, and biological inference. For subject level integration, we may distinguish between vertical and horizontal integration [58]. In vertically integrated studies, multiple levels of omic data are gathered on the same subjects. Vertical integration forms the basis for most of integrative approaches discussed above (e.g., metaboQTLs) [15] and can be applied with all of the aforementioned study designs (Figure 3). In horizontally integrated studies, the integration may occur using information derived from biosamples from a different study population(s); direct integration is not possible, but the findings from the primary omic can be “conceptually integrated” with complementary information derived from the external omic analysis. For example, known protein interactions with a given metabolite identified to be disease-associated from a metabolomic analysis may provide deeper insights into underlying mechanisms, and lend further plausibility to the finding itself. Horizontal integration may provide corroborating evidence to support the initial analysis without directly replicating it. 

During analysis, the form of integration may be divided into sequential versus joint categories. Sequential integration occurs when there are multiple sequential steps to integration. Each omic datatype is analyzed in succession, where the subsequent steps/analyses are dependent on the findings from the former steps/analyses [59]. When attempting to integrate multi-omics data from the same samples or subjects it may make sense to adopt a central dogma-based framework to establish assumptions about directions of temporal effect (e.g., genome/transcriptome/proteome/phenome). This is in contrast with direct joint integration which occurs when individual level omic data from multiple source populations are combined into a single matrix (before or after dimension-reduction) for simultaneous analysis. In this case, great care needs to be taken to address differences in scale and variance of data from different platforms so that the results are not dominated by one class of omics data. This is especially salient for metabolomics integration: metabolomics as a field is still developing, and the number of catalogued, known metabolites is quite low relative to other omics. Furthermore, the identification of new small molecules and the establishment of high-quality reference standards remain laborious and time-consuming. Multi-block modeling methods offer one approach to address concerns of scale in a principled manner.

Finally, the limits of biological inference are best characterized by the distinction between data-driven vs knowledge-driven integration. Data-driven integration relies on statistical integration of the molecular data based on metrics such as significant associations, or clustering and co-expression. Data-driven approaches do not incorporate underlying biological knowledge so most often additional work is necessary to correctly understand the findings that are observed. In contrast, knowledge-based integration relies on validated or expected disease, biological, and chemical annotations for known analytes (genes, proteins, metabolites, microbes, etc.). However the strength of knowledge-based integration is also its weakness—this form of integration relies on what is already known, and thus is particularly useful for identifying potential mechanisms of disease action, but may also penalize novel multi-omic findings if information on either level of omic data is not annotated in the reference database used for analysis. 

### 3.1. Data-Driven Methods

#### 3.1.1. Correlation-Based

Information on molecular interactions can be extracted directly from omics data. This allows us to infer relationships in vivo from the observed system, obtaining computed associations specific for the considered species, subgroup, tissue and omics. In the following section, we discuss the most popular methods to estimate pairwise associations in omics data analysis. These can also be conveniently visualized and analyzed systematically in the form of networks, where nodes represent variables and the edges between them their associations. 

One of the most common approaches to determine whether two variables are related is to compute their Pearson correlation coefficient, which is a measure of linear association between a pair of normally-distributed variables [60]. This association measure is widely used in omics data analysis [61,62,63,64,65,66,67,68]. Non-parametric correlation coefficients can be computed using Spearman’s rank correlation [69] instead of Pearson. This approach is more robust to outliers and can identify any monotonic relationship between variable pairs. However, it does not account for the magnitude of the expression variation, but only for the difference in ranks and is therefore less common in omics data analysis [70,71]. For non-monotonic, non-linear associations, or associations based network topology, or non-metric similarities measures like the Mutual Information (MI), Distance Correlation, topological coefficient or stochastic embedded neighborhood are considered by some authors [72,73,74,75]. 

Notably, Pearson correlation does not account for the presence of confounding effects. This problem can be overcome by using partial correlation, which accounts for the presence of confounders by regressing out the effect of such variables from the correlation coefficient [76]. Networks from full-order partial correlations, i.e., partial correlations corrected for all other variables as confounders, are also known as Gaussian Graphical Models (GGMs). In the case of fewer samples than variables (n < p), regularization approaches for the estimation of partial correlation are necessary, for example using shrinkage methods as implemented in ‘GeneNet’ [77] or L1 regularization as in the Graphical Lasso [78]. GGMs have been shown to be a powerful tool to identify direct biochemical synthesis reactions in mass-flow systems like metabolomics [79,80,81,82,83] and glycomics data [84]. Other applications of this approach include the inference of genomics networks [77,85,86,87], transcriptomics networks [88,89] and gene-expression networks [90,91,92], as well as multi-omics networks [28]. This approach also assumes a multivariate Gaussian distribution and might therefore not be suited for non-normally-distributed data. Binary and categorical response variables can be included by employing a Mixed Graphical Model (MGM), which have found applications in genomics [93], gene-expression [94,95,96] and multi-omics data analysis [97]. 

#### 3.1.2. Networks/Topological Structure

The network representation of molecular associations has been proven to be a compelling visualization to better understand the relationship between the different omics layers. Once an interaction network has been established, either from the data or from external resources, various approaches can be applied to systematically extract relevant information at different granularities. For example, the network topology can be exploited to identify of sets of nodes, or modules, that are highly connected within themselves but have few connections with the rest of the network. The underlying idea is that, given their connectivity, molecules within a module are likely to be functionally related and might therefore represent a more biologically meaningful unit than a single metabolite or gene [98,99,100]. Modules can be defined with a wide variety of approaches: popular ones include Newman’s community detection [101], and WGCNA [102], but many other network clustering approaches are available, see Mitra et al. [103] for a review. Modules can also be identified by maximizing the association to a given phenotype [104,105,106] and provide an interesting joint readout of how different omics interact among themselves or in relation to an outcome of interest.

#### 3.1.3. Bayesian Networks 

Bayesian Networks (BN) are a machine learning method that organizes variables into a network, and then uses Bayesian statistics to compute likely values of modeled variables. BNs can be used to build predictive models of case/control status [107]. For building predictive models, a BN has several benefits vs other predictive modeling frameworks: (1) the ability to cleanly model nonlinear interactions between variables; (2) models are easily interpretable due to the network connections between variables; (3) the parameters of the Bayesian priors can be used to protect against overfitting and improve the replication of predictive performance; (4) clean integration with other ancillary machine learning techniques such as cross-validation, bootstrapping, and permutation testing [108].

When performing BN modeling on an integrative dataset, or indeed any dataset, the modeling occurs in two steps. First, a BN structure must be obtained. This is the network structure which reflects the statistical dependence among variables present in the dataset. An iterative heuristic search across the network space is conducted starting with an initial network. Small changes are added to the network until further changes seem unhelpful. A good network structure is one that represents the dataset well; more precisely, one that maximizes the posterior likelihood of the dataset, according to the Bayesian statistics defined by the network. Once a BN structure has been defined, the second part of a BN analysis is to predict the phenotype using the values of nearby, connected variables to infer the most likely values for the outcome. The resulting BN can be applied on the same dataset that was used to discover the BN structure to assess model fit; or it can be applied in a replication cohort to demonstrate validity and generalizability of the BN. 

Historically, BNs deal with only binary variables, or categorical variables. For continuous data, a Conditional Gaussian Bayesian Network (CGBN) [109] is required. This approach combines categorical variables with normally-distributed continuous variables within the same Bayesian statistical framework and network framework. With a CGBN, other omic variables can be easily handled: metabolite concentrations, mRNA concentrations, etc.; as well as other demographic data. 

Obtaining the BN network structure is essentially a variable-selection process, thus concatenation can be used when integrating multiple omic datatypes for CGBN analysis [110]. BNs are computationally intensive, and have trouble handling many thousands of variables. Variables can be pre-filtered by assessing their statistical association with the main outcome of interest, measured by the Bayes Factor (BF) [111] (threshold is typically BF > 0). Available BN software (CGBayesNets [112]) contains routines for doing this simply. Bayesian Networks, and CGBNs in particular, are well suited for building predictive models of clinical outcomes from integrative omic datasets. One of the challenges in construction of Bayesian networks is that the most probable network is one of many near-equally likely networks, each potentially with a different topology and directionality. Therefore, the uncertainty in the final BN should be clearly noted or illustrated in any report. Because BNs are not acyclic in their directionality, they do not necessarily converge to reveal the true underlying causal mechanism, and thus should not be interpreted as causal. BNs are suited for building prediction models, but caution is recommended in any interpretative exercise for the final model.

#### 3.1.4. Regression Approaches

Regression analyses are the most ubiquitous analytic strategy in molecular epidemiology, and can be employed whether the context is inference and effect estimation, or classification/prediction. In inference and effect estimation, regression techniques may be used to test hypotheses of association between a given omic variable (or variable set) with a phenotype of interest, and also to specifically estimate the magnitude of association between the two. A familiar use of regression techniques in multi-omic analyses is that of the SNP-metabolite association study [15,113]. Other useful approaches that have been applied in other integrative omic studies, but which have not yet seen wide-spread adoption in the integrative metabolomics literature, include Mendelian randomization (MR) [114,115] and causal mediation approaches [116,117,118,119]. Mendelian randomization techniques facilitate inference of causal effects using an instrumental variable technique, wherein genetic variants are treated as instrumental variables (i.e., variables that exert effect on a phenotype of interest *only* through their effect on the primary exposure of interest). To illustrate the use of this approach in a metabolomic context, with genetic variants as instruments, the assumptions necessary to make a valid inference of causal effect would include: (1) the genetic variants are causally associated with a given metabolite (i.e., a metaboQTL), (2) the genetic variants are causally associated with the phenotype of interest, and (3) the genetic variants are independent of unobserved confounders of the metabolite-phenotype relationship after adjusting for known confounders [114]. This last condition is trivially met if the assumption that genetic variants are established at birth and therefore not subjects to confounding by environmental and behavioral factors later in life. In analyses such as these, it should theoretically not be necessary to understand the complete integrated SNP-metabolite network, so long as we are confident that MR assumptions are reasonable. In causal mediation analyses with multi-omic data, the usage is similar to that seen in genome-wide association scans, but the straightforward regression model is replaced with a two stage mediation analysis framework, in which sites are interrogated one at a time for evidence of mediation of the association between an exposure and phenotype of interest by an omic marker. Examples of such omic-wide mediation analysis scans were first seen in epigenetics literature [120,121], but would be appropriate in the metabolomics context as well.

An example of predictive regression modeling is partial least squares (PLS). PLS is similar to principal component analysis, although the components in PLS are selected to explain maximum covariance between predictors and the response (or a linear combination of responses). To reduce the size of the input data, which can reach many tens of thousands in multi-omics integration, sparse PLS methods can be applied where sparsity constraints from Lasso penalization are used for simultaneous feature selection and integration. Another popular extension of PLS used in multi-omics integration is two-way orthogonal PLS (O2-PLS) [122,123,124], which models the predictive and systematic variation. To accomplish this, the variation is decomposed into three parts: (1) the joint variation that represents the covariance of between the two omic data types; (2) the orthogonal variation that represents the systematic variation; (3) the variation due to noise. While the O2PLS bi-directional model is limited to 2 omics datasets, the method has been expanded to incorporate multi-omics in OnPLS [125,126]. This projection method simultaneously models multiple data matrices, reducing feature space without relying on a priori biological knowledge. Other approaches developed to separate the variation in multi-omic data sets into joint variation, local variation (systematic variation present in only a subset of the data sets) and distinct variation (systematic variation only present in a single data set) include Joint and Individual Variation Explained (JIVE) [127], simultaneous component analysis with rotation to common and distinctive components (DISCO-SCA) [128], Structural Learning and Integrative DEcomposition (SLIDE) [129], and penalized exponential family simultaneous component analysis (P-ESCA) [130]. Because PLS approaches are susceptible to overfitting, which is of particular concern when the intent is to predict phenotype, it is important to validate models using approaches such as cross-validation, and preferably also in an independent dataset, and to report final models with all relevant metrics that provide an assessment of model robustness [131].

It is worth noting that the regression-based methods described so far may be driven by variables that have the largest variance, and can be insensitive to low-variance variables. This point is of particular importance in multi-omics integration applications, where the variance of features can differ greatly both within and between a given omic data type. Therefore, data should be scaled as appropriate for the research question at hand (e.g., weighting by the inverse variance of each omic feature might be appropriate if the goal is to keep any given feature from dominating the others).

Computational frameworks such as Mixomics [132] provide access to a multitude of multivariate methods but do not include knowledge-based methods and require statistical and computational knowledge. Other tools perform specific numerical analyses, such as DiffCorr [133] for identifying global correlations, and IntLIM [134] for identifying phenotype-specific associations leveraging data from multiple omics.

### 3.2. Knowledge-Driven Methods

#### 3.2.1. Reference Databases

Molecular interactions and relationships between molecules are typically obtained from publicly available resources. There are relatively few databases that make it possible to link metabolomics data to other omics layers, and they usually focus on describing metabolic reaction networks at genome-scale. The most common and extensive resources for metabolomics research include manually-curated databases such as KEGG [135], Reactome [136], WikiPathways [137], and Recon [138]. Due to its extensive manual curation and large community, Recon is the most complete database on human metabolism to date: it collects detailed information from literature and validation experiments about biochemical reactions in a variety of metabolic pathways and allows direct linkage of metabolites to, among others, proteins and genes involved in such reactions and pathways.

#### 3.2.2. Pathway-Based Analysis and Multi-Omic Set Testing

Pathway enrichment approaches can be used to assess whether an overall pathway, rather than single metabolites or molecules, is differentially regulated in two or more experimental conditions. Although various tools exist to perform pathway enrichment on metabolomics or single omics data alone [139,140,141,142,143], new methods that allow the integration of multi-omics datasets are becoming available [144,145,146] and exploit the common pathway ontology of multi-omics data provided by publicly available resources. Once a list of metabolites and other omic targets of interest have been identified, their biological relevance can be assessed by inputting these lists into software tools such as MetaboAnalyst [139,147], PaintOmics [148], IMPaLA [144], MetaBox [149], and RaMP [146]. Notably, while knowledge-based approaches are instrumental in interpreting the molecular data, they fall short when uncovering new knowledge because many annotations are yet to be discovered, particularly for metabolites.

Provided that associations between metabolites and other omic features are known, it may be of interest to test the association or joint effect of an entire pathway, or to conduct agnostic searches for trait associations across biological sets. In these approaches, mappings between the metabolites and the molecules or variants with which they interact are critical, and the metabolite-molecule pairings are tested as a set. Several techniques for biological set testing have been developed for multi-omic contexts which leverage combining/meta-analytic [144], summary statistic or rank-based enrichment/over-representation analysis [144,150,151], network-based topology [152], joint effect hypothesis testing [153], and high-dimensional causal mediation [154,155], While not all of these approaches were developed for metabolomic data, most are easily extendable to metabolomic contexts provided that the appropriate mapping is available between the two omic types. All of these set testing methods depend, however, on mappings between metabolites and their interacting molecules/variants a priori. For example, the largest metabolite annotation database, the Human Metabolome Database (HMDB) contains entries for 114,100 metabolites yet only ~22% are mappable to pathways [146,156]. The use of numerical and knowledge-based -omics integration methods that adopt and combine both approaches would thus allow users to maximize known and novel relationships in their data.

#### 3.2.3. Constraint-Based Modeling: Flux Balance Analysis

Flux balance analysis (FBA) is a constraint-based modeling approach widely used in genome-scale metabolic network reconstruction [46]. In FBA, the mathematical representation of metabolic reactions relies solely on the stoichiometry of chemical reactions, which impose constraints on the flow of metabolites through the network at steady state (the total amount of output metabolites must be equal to that of input metabolites). Additional constraints such as lower and upper bound on the rates/fluxes of individual reactions, or maximum rate of substrate uptake can also be imposed. Within these constraints, FBA uses linear optimization to solve for fluxes that maximize or minimize a defined phenotype (objective function) [46]. Detailed manual curation of all known metabolic reactions and genes encoding enzymes in a biological system is necessary for FBA-based metabolic network reconstruction, which in itself is an integration of genetic and metabolic information. In humans, several such efforts have been undertaken, including Recon [138], the Edinburgh Human Metabolic Network [157], and the Human Metabolic Reaction [158].

Extending constraint-based modeling methods such as FBA from microorganisms to multi-organ systems (e.g., human) is complicated by the fact that different tissues have varying and largely unknown metabolic functions and rates of metabolite uptake and secretion [159]. The integration of other omic data types into FBA can be informative of the definition of objective functions and constraints put on the metabolic reactions. Several FBA-based methods for integrating transcriptomic data with genome-scale metabolic network reconstruction have been reviewed previously [160]. Integration of tissue-specific gene expression and proteomic data into a global human metabolic network has been used to characterized different metabolic behaviors in ten tissue types [159]. This is an example of horizontal integration in the sense that data on tissue-specific gene expression and protein abundance were obtained from publicly available databases, and were used to inform tissue-specific enzymatic activities.

FBA can also be used in the study of human gut microbiome, which requires extensive curation of metabolic reactions and enzymes known in human microbiota. AGORA (assembly of gut organisms through reconstruction and analysis) is one such resource of genome-scale metabolic reconstructions semi-automatically generated for 773 human gut bacteria [161]. Using publicly available metagenomic data mapped to AGORA, personalized metabolic models of the microbial community were constructed accounting for strain-level abundances, and the metabolic potential in individual microbiome can be assessed [162]. One well-established and commonly-used computational tool for FBA-based analyses is the Constraint-based reconstruction and analysis (COBRA) Toolbox in MATLAB [163]. Despite its popularity, FBA-based optimization results require experimental validation, which can be hard to obtain in human populations, and are only suitable for characterizing fluxes at steady state [46]. Modified forms of FBA have been developed to take into consideration kinetics and regulation on enzyme activities, and adapt to dynamic network models [164,165].

### 3.3. Strategies for Type I Error Protection in Multi-Omics Analyses

Because of the wide range of data types, research objectives and analysis options in multi-omics projects it is virtually impossible to offer a single strategy to protect against false positive/negative inferences. Nevertheless, whenever considering such high-dimensional problems where many tests of association are considered or many features are aggregated or ranked it is important to explicitly address the possibility that some results are due to chance. For analyses that produce a parametric test statistic and associated *p*-value, a common approach is perform a permutation procedure to simulate the null hypothesis thousands of time in order to estimate the likelihood of a false positive result. Variations on this procedure can be used to determine the “effective number of tests” to be used for a corrected Bonferroni type of p-value adjustment [166]. The advantage of this approach is that it explicitly preserves and accounts for correlational structure of the omics data under consideration. The Benjamini–Hochberg False Discovery Rate (FDR) [167] is another less rigorous but much simpler strategy to provide an estimate of the likelihood of a false positive result. Another strategy to consider is some form of cross-validation of bootstrapping to determine the extent to which observations are robust to variations in the sample set used for analysis. An attractive feature of this approach is that it can be applied to other types of analysis results (such as network composition) that are not amenable to conventional parametric statistics. All of these approaches are some form of internal validation. However, ultimately the best assurance that any multi-omics analysis result is true is to replicate the result in an independent experiment or analysis of unrelated samples, specimens, or individuals.

## 4. Conclusions

Multi-omic integration in epidemiological studies represents a significant opportunity to increase the understanding of the underpinnings of health and disease in terms of biological mechanism, therapeutic targets and biomarkers [18]. In order for this potential to be realized, careful design and analysis considerations are critical. In this review, we provide a framework for thoughtful multi-omic study design and analysis that is guided by the initial multi-omic question (Figure 2). The inferences drawn from a multi-omic question rely on both the study design and biosampling schema. Longitudinal studies facilitate prognostic prediction or estimation of causal effects between the metabolome, other omes, and a phenotype, while cross-sectional studies are limited to identifying associations or prevalent disease classification. We review several longitudinal study designs that consider possible relationships between metabolites, another omic, exposure, and phenotype to answer different multi-omic research questions. In addition to study design, the role of sampling schemas for multi-omic data is also discussed for informing inference and analyses. Study designs that involve repeated sampling at the same time point on a defined set of study subjects to generate multi-omics are particularly useful for inference.

We review several important omic-specific considerations that may facilitate integration with the metabolome. For example, the identification of metabQTLs and GIMs relies on the premise that the genome precedes the metabolome. Other considerations may be more focused in omic data generation (for example. both proteomic and metabolomic data may be generated in the same laboratory, which may reduce several sources of variation and facilitate integration).

The multi-omic question is the most important component of the analysis, beginning with whether the analysis will be focus on effect estimation or prediction/classification. The analytical approaches to integration are either data-driven or knowledge-driven; however, both have limitations. Data-driven integration relies on statistical metrics (e.g., significance or distance) and while they are able to identify novel disease features or associations, they may not incorporate biological knowledge. In contrast, knowledge-based integration relies on what is already known, and thus is particularly useful for identifying potential mechanisms of disease action, but may encounter challenges in the identification or prediction of novel disease risk factors.

The most appropriate analytic strategy will depend on the study design, types of omic data available, and target of inference of the study, as the assumptions underlying the methods discussed above vary, and each method accounts for different subsets of statistical concerns. While the analytical strengths of one approach may address the weaknesses of another, the most comprehensive analysis may incorporate multiple analytical methods. Although not discussed in detail, we also emphasize that critical to the success of any of the analytic approaches presented in this review is the quality of the acquired data. To detect subtle systemic shifts in biological pathways in heterogeneous diseases across multiple omics data blocks, it is necessary to have omics data with a high level of precision and robust QC protocols that minimize non-biological technical variation that might obscure true signals. Further development of integrative methods and thoughtful incorporation of study design principles is necessary to continue improving the individual and systems-level understanding of risk-conferring biological processes.
